# Drug Targeting of α-Synuclein Oligomerization in Synucleinopathies

**Published:** 2008-04-10

**Authors:** Tiago Fleming Outeiro, Aleksey Kazantsev

**Affiliations:** 1 Instituto de Medicina Molecular, Instituto de Fisiologia, Faculdade de Medicina, Universidade de Lisboa, Av. Prof. Egas Moniz, 1649-028 Lisboa, Portugal; 2 MassGeneral Institute for Neurodegenerative Disease, Harvard Medical School, CNY114 16th St., Charlestown, MA 02129, U.S.A

**Keywords:** Parkinson’s disease, drug discovery, quality control systems, aging, α-synuclein, synucleinopathies, aggregation, neurotoxic oligomers

## Abstract

The heterogeneity of symptoms and disease progression observed in synucleinopathies, of which Parkinson’s disease (PD) is the most common representative, poses large problems for the discovery of novel therapeutics. The molecular basis for pathology is currently unclear, both in familial and in sporadic cases. While the therapeutic effects of L-DOPA and dopamine receptor agonists constitute good options for symptomatic treatment in PD, the development of neuroprotective and/or neurorestorative treatments for PD and other synucleinopathies faces significant challenges due to the poor knowledge of the putative targets. Recent experimental evidence strongly suggests a central role for neurotoxic α-synuclein oligomeric species in neurodegeneration. The events leading to protein oligomerization, as well as the oligomeric species themselves, are likely amenable to modulation by small molecules, which are beginning to emerge in high throughput compound screens in a variety of model organisms. The therapeutic potential of small molecule modulators of oligomer formation demands further exploration and validation in cellular and animal disease models in order to accelerate human drug development.

## Introduction

Protein conformational disorders are a group of diseases associated with the misfolding and aggregation of one or more proteins. They are believed to result from the inability of proteins to be functional which, in some instances, is associated with the accumulation of abnormally folded proteins. Proteins, as the main effectors in the cell, play underpinning roles in all biological processes. Therefore, it is not surprising that the list of these diseases is continuously expanding, as new proteins are identified and their functions understood.

Despite the well-known connection between protein misfolding, aggregation, and disease, the manner by which misfolding results in disease is not clearly understood. In some cases, it seems that the deposition of protein aggregates may physically disrupt the functioning of specific cell groups and the respective tissues and organs where those cells are located. In other cases, it seems that the lack of functional protein, due to its recruitment into the aggregates, results in the failure of crucial cellular processes (Thomas et al. 1995). However, for neurodegenerative diseases, such as Alzheimer’s, Parkinson’s or the Prion diseases, it appears that the symptoms arise from the destruction of cells by a “gain of toxic function” that results from the aggregation process (via oligomers, protofibrils, amyloid fibrils or other intermediates) or by a combination of both this gain of toxic function and a loss of normal function of the protein (Ross and Poirier, 2004; Caughey and Lansbury, 2003).

## Synucleinopathies

α-synuclein (aSyn), a member of the synuclein family of proteins, was initially identified on the electric lobe of *Torpedo californica* for reacting to antiserum against purified cholinergic vesicles (Maroteaux et al. 1988). The synucleins became connected to several neurodegenerative disorders after the initial report of a non-Aβ component of AD amyloid (NAC), consisting of a 35 amino acid polypeptide generated by cleavage of aSyn.

The *PARK1* locus, which encodes for aSyn, became associated with Parkinson’s disease (PD), when a point mutation was found in an Italian kindred afflicted by autosomal dominant PD. The mutation causes a threonine for alanine substitution at position 53 (A53T) (Polymeropoulos et al. 1997). This discovery was then followed by a report identifying aSyn in Lewy bodies (LBs), concentric hyaline cytoplasmic inclusion bodies. All LBs were shown to contain the protein aSyn (Spillantini et al. 1997; Irizarry et al. 1998).

Shortly thereafter, another familial form of PD was linked to a mutation in aSyn causing a proline for alanine substitution at position 30 (A30P) (Kruger et al. 1998). More recently, a third mutation consisting of a lysine for glutamate substitution at position 46 (E46K) was discovered to be associated with familial PD (Zarranz et al. 2004). Additionally, duplications and triplications of the *PARK1* locus have also been linked to familial PD. The dominant nature of the inherited mutants is thought to reflect a gain rather than a loss of function in the aSyn proteins.

After the initial discovery of aSyn in LBs in PD, the protein was detected in cellular inclusions in several other neurodegenerative diseases including dementia with Lewy bodies (DLB), multiple system atrophy (MSA), and Hallervorden-Spatz syndrome, now called neurodegeneration with brain iron accumulation type 1 (NBIA). The neurodegenerative diseases that share aSyn pathology as a primary feature are collectively known as synucleinopathies.

PD is one of the most common progressive, neurodegenerative disorders affecting about 2% of people over 65 years old and 4%–5% of people over 85 (between one and one-and-a-half million Americans). PD is characterized by loss of dopaminergic neurons in the *substantia nigra pars compacta* and is accompanied by muscle rigidity, bradykinesia, resting tremor and postural instability.

It is now known that LBs can be seen in pigmented neurons of the *substantia nigra* in almost every case of PD (Jellinger, 1987). However, LBs have also been observed in the brains of asymptomatic individuals (Nussbaum and Polymeropoulos, 1997). The intracerebral formation and spreading of LB pathology has been extensively studied. LBs and Lewy neurites appear at defined induction sites and pathology advances in a topographically predictable sequence. As PD progresses, components of the autonomic, limbic, and somatomotor systems become particularly badly damaged. During presymptomatic stages 1–2, inclusion body pathology is confined to the medulla oblongata/pontine tegmentum and olfactory bulb/anterior olfactory nucleus. In stages 3–4, the substantia nigra and other areas of the midbrain and forebrain become the focus of pathological changes. When this happens, individuals start to display the first disease symptoms. In the end-stages 5–6, the process enters the neocortex, and the disease manifests itself in all of its clinical dimensions.

### α-Synuclein misfolding, aggregation, and toxicity

The ‘amyloid hypothesis’ (developed originally for Alzheimer’s disease) states that the aggregation of proteins into an ordered fibrillar structure is causally related to aberrant protein interactions that culminate in neuronal dysfunction and ultimately neurodegeneration (Hardy and Selkoe, 2002). The actual nature of the toxic species is, however, presently unknown. Evidence from PD suggests the hallmark inclusions (LBs) may actually be a protective mechanism neurons developed to preclude the accumulation of the pathogenic intermediates, which have been proposed to be aSyn oligomers (Ding et al. 2002).

Aggregation of aSyn and the putative gain of toxic function may contribute to haploinsufficiency by entrapping the wild type protein, thus reducing the amount of available functional aSyn. Recent studies in yeast suggest that once aSyn starts to aggregate it may recruit aSyn away from other sites in the cell, harmonizing the gain and loss of function hypotheses (Outeiro and Lindquist, 2003). One hypothesis is that molecular crowding could first accelerate the formation of aSyn spherical protofibrils, a process that involves β-sheet formation and then chain-like and annular protofibrils (Ding et al. 2002). Other studies show that crowding also dramatically accelerates aSyn fibril formation. *In vitro* studies with purified aSyn demonstrated that protofibril and fibril formation require different critical concentrations, suggesting there are ranges under which one may form preferentially over the other. If annular protofibrils do indeed exist in vivo, they could exert toxicity because of their binding and permeabilization of vesicles. These pore-like structures formed, in vitro, by aSyn have been visualized by electron microscopy (Lashuel et al. 2002b; Lashuel et al. 2002a) and by atomic force microscopy (Ding et al. 2002) and they are structurally similar to a subset of Aβ protofibrils and other unregulated pore-forming toxins (Volles et al. 2001; Lashuel et al. 2002b; Lashuel et al. 2002a).

Recent findings in cellular systems are consistent with the toxic protofibril hypothesis. The accumulation of prefibrillar aggregates in the membrane fraction, prior to the appearance of aSyn inclusions, was associated with Golgi fragmentation and a reduction in cell viability (Gosavi et al. 2002). Lysosomes may also be disrupted by aSyn protofibrils in a similar fashion. If lysosomal degradation of aSyn is critical in aSyn turnover this could start a vicious toxic cycle (Stefanis et al. 2001).

In PD flies, the disconnection between pathogenesis and aSyn inclusion formation adds support for roles of aSyn oligomers rather than fibrillar inclusions in toxicity and pathogenesis (Auluck et al. 2002).

Notwithstanding the recent findings that favor roles for aSyn oligomers in cellular toxicity, it is also likely that fibrillar and/or amorphous aSyn aggregates also contribute to physical damage in neurons (Neumann et al. 2002).

Several groups described differences in the aggregation kinetics of three different forms of aSyn (WT, A53T and A30P) *in vitro* (Conway et al. 1998; Conway et al. 2000; Li et al. 2002; Narhi et al. 1999; Ostrerova et al. 1999; Serpell et al. 2000). Structural differences between these aSyn alleles have also been reported although the crystal structure of the protein is unknown (Bussell and Eliezer, 2003). aSyn belongs to the family of ‘natively unstructured proteins’ because it lacks defined secondary structure under physiological conditions. This property of aSyn, which makes it different than most other drug targets, is likely to constitute a major challenge for drug discovery efforts. Nevertheless, it is known to adopt α-helical conformation upon lipid binding or in the presence of detergents.

All four forms of aSyn (WT, A30P, E46K, A53T) have the ability to form fibrils with the typical properties of other amyloids. However, the A30P mutant seems to form fibrils slower than WT aSyn, whereas the A53T fibrillizes faster than WT (Conway et al. 2000). These findings are suggestive of a toxic role for small aSyn oligomers (not the fibers themselves) in disease. The A30P mutant, unlike A53T and WT aSyn, seems to be defective in binding to vesicles and membranes (Jo et al. 2002; Jensen et al. 1998). In cell culture, the conformation of the A30P mutant also differs from that of WT and A53T aSyn (McLean et al. 2001). These differences also point at disease mechanisms that might differ depending on the mutant form of aSyn. The corollary to this is that distinct intervention strategies might be necessary depending on the mutation present.

### Protein misfolding, cellular quality control systems as targets for therapeutic intervention in neurodegenerative diseases

Protein aggregates characteristic of AD, PD, prion diseases, and other neurodegenerative diseases share common morphological and biochemical features. In addition, they co-localize with several of the same proteins, including ubiquitin, proteasome, and lysosome subunits, and molecular chaperones, which collectively constitute the protein quality control (QC) systems in the cell (Muchowski and Wacker, 2005). This sequestration of the cell’s QC machinery with inclusions might lead to a loss of function, rendering the cell less likely to refold/degrade other misfolded/aggregated proteins, causing a series of events that may ultimately result in cell death.

An alternative possibility is that, with aging, environmental insults, mutations or other unidentified triggers, the activity of the QC systems becomes compromised causing aggregation prone proteins to misfold and be left “unattended”, leading to their accumulation and the consequent pleiotropic effects, including cell death.

A firm proof on involvement QC system in neurodegeneration came from genetics of familial PD cases (Hardy et al. 2006; Ross and Pickart, 2004; Dawson, 2006; Ardley and Robinson, 2004). Causative mutations have been identified in Parkin and UCH-L1 genes, encoding a ubiquitin E3 ligase and a ubiquitin hydrolase, respectively. Recessive and dominant traits of inheritance for these genes have been determined for familial cases of PD, caused by mutation in Parkin and in UCH-L1. Homozygous mutations in the DJ-1 gene product have been described recently in two families with autosomal recessive PD inheritance. DJ-1 is likely a redox-sensitive molecular chaperone, activated in an oxidative cytoplasmic environment. Mutations in DJ-1 are responsible for altering the cellular response to oxidative stress and proteasome (Shendelman et al. 2004). There are some evidences suggesting that genetic defects in gene products associated with degradation pathways cause abnormal aSyn turnover and subsequent formation of oligomers.

**Figure 1:**
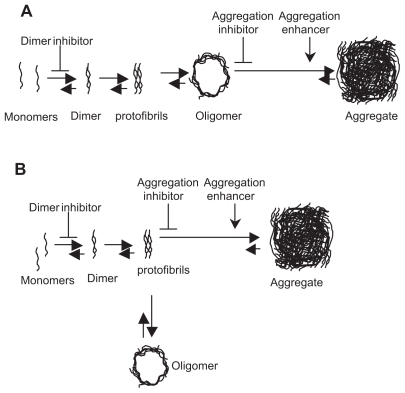
Modulation of aSyn aggregation pathway with small molecules is aimed at reducing the levels of oligomeric species. The two possible models of aggregation described, where oligomeric species are **A**) true aggregation intermediates, **B**) bypass products. In both models, aggregation enhancers minimize oligomer concentration by shifting the equilibrium towards the formation of aggregates. Dimerization inhibitors prevent the subsequent steps on the aggregation pathway, including oligomerization. Irrespectively from the model, aggregation inhibitors may cause an increase in the concentration of oligomers either **A**) directly, or **B**) indirectly, by shifting the equilibrium towards the formation of protofibrils. Aggregates are clearly not reliable phenotypic markers to identify agents preventing oligomerization in cell-based screens.

Thus, identifying and targeting the misfolded state of each of the proteins involved in these disorders holds promise to constitute a useful therapeutic strategy (Fig. 1). It will, therefore, be important to continue to develop cell models which recapitulate the molecular mechanisms of disease and where the conformation of these proteins can be readily assessed and manipulated. A variety of such models, from yeast to primary dopaminergic neurons are already proving to be very valuable “living test tubes” for these types of studies due to their great ease of manipulation (Outeiro and Muchowski, 2004; Outeiro and Giorgini, 2006; Cooper et al. 2006).

### Targeting neurotoxic oligomers

The stabilization of the native structure of proteins, preventing their initial misfolding, is an appealing intervention strategy. For oligomeric proteins, such as transthyretin, for example, compounds capable of stabilizing the functional tetrameric structure hold great potential and are in the process of being developed. This approach would prevent the formation of the toxic intermediates and aggregated species and would therefore be beneficial.

Recent experimental evidence suggests the formation of neurotoxic oligomers is a key pathological event not only in PD but also in other synucleinopathies. Aggregation of misfolded proteins appears to be a complex physical-chemical process, superimposed in the cellular environment and modulated by various cellular components. The process of protein aggregation consists of two major phases: a rate-limiting nucleation phase and the subsequent rapid polymerization phase. During fibrilization, soluble and aggregated species exist in equilibrium, which becomes greatly shifted towards the latter. Soluble oligomers may be fruitful aggregation intermediates which, by operational definition, have increased affinity for fibrils. One possibility is that the process of joining the aggregate diminishes the neurotoxic function(s) of oligomers. Alternatively, oligomers could constitute bypass products of aggregation, and then exist in equilibrium between monomers and fibrils (Fig. 1). The equilibrium between soluble and aggregated species can be shifted towards the former or the latter by using small molecule inhibitors or enhancers of aggregation, respectively. Both these models suggest that enhancers of aggregation promote formation of large inclusions, limiting the presence of all soluble species, including oligomers. The strategy of using aggregation inhibitors, however, aiming to increase the levels of soluble monomers which can be readily subjected to degradation by proteasomes and/or authophagy, may not be advantageous for diseases where the QC system is impaired. Thus, promoting the formation of large inclusions appears as a safer and, potentially, more beneficial strategy for synucleinopathies and other protein aggregation disorders. Therefore, it is important to consider possible molecular targets modulating the process of inclusion formation, such as enzymes responsible for post-translational protein modification, proteins implicated in aggresome formation such as HSP70 and HDAC6, a sub-set of chaperones responsible for protein re-folding (HSP70, HSP27, HSP90), and proteins mediating the seeding and transport of misfolded proteins to the nucleation sites (Iwata et al. 2005; Bandhyopadhyay and Cuervo, 2007). In addition, enzymes modifying cytoskeleton and microtubule components and the affinity of cellular protein complexes to monomers, oligomers, and aggregates, may also be found to be important regulators of inclusion body formation (Niewiadomska et al. 2006; Dimakopoulos, 2005; Lee et al. 2006; Feng, 2006).

### Drug discovery strategies

The rationale for targeting components of the QC systems is based on solid evidence from genetics and pathophysiology. While extensive studies support a major role of molecular chaperones in synucleinopathies as well as in other neurodegenerative diseases, neither these QC components nor those which gene products are defective in familial PD are easy drug targets for small molecules. Individual molecular chaperones play multiple roles in the cell and work in a finely orchestrated fashion to execute different cellular responses (Macario and Conway de Macario, 2005).

Thus, rationalizing and prioritizing selection of chaperones as targets is not a trivial approach. Notwithstanding, *in vitro* assays have been designed to identify small molecule modulators of chaperone activities. These assays are based on the re-folding of heat-inactivated luciferase in the absence or presence of candidate chaperones. A simple luminescence readout detecting the recovery of the native conformation by luciferase, allows screening the large compound libraries in a high throughput manner.

The drug discovery process for synucleinopathies has been delayed over the years by the elusive nature of the mechanisms leading to neurodegeneration and, subsequently, by the lack of useful molecular drug-targets. As a major effort to overcome these difficulties several “black box” assays have been developed. These are usually cell-based assays which try to recapitulate phenotypic and functional aspects of disease. This alternative unbiased approach, which does not require any assumptions in terms of the molecular targets and mechanisms involved, led, through chemical and genetic screens, to the identification of both small molecules and also potentially novel drug-targets.

While “black box” assays were successful to identify specific hits in chemical screens, hit-optimization for potency, selectivity and ADMET based on primary “black box assays”, has not been as straightforward as initially thought. Therefore, it is essential to identify the molecular targets of the identified compounds in order to expedite development pre-clinical candidates.

### Assay development

Several cell-based assays targeting key molecular aspects of disease, such as aSyn cytotoxicity, aggregation, or proteasome have been developed. For example, overexpression of wild type aSyn in mammalian cells of neuronal origin causes significant cytotoxicity and aggregation of misfolded protein. The design of this model was to mimic the genetic multiplication of aSyn alleles as seen in familial PD cases (Singleton et al. 2003), resulting in a 2–4 fold-increase in the levels of aSyn. This cell-based model has been employed for the validation of compounds rescuing cytotoxicity by inhibiting or promoting inclusion formation (Bodner et al. 2006). Subsequent assay development was focused on early event in formation of oligomers, such as aSyn dimerization and oligomerization (Klucken et al. 2006). These next generation assays were FRET-based or employed readouts which allow the detection of protein-protein interactions in the cell.

## Screening Platforms

### Yeast cells

The budding yeast *Saccharomyces cerevisiae* is a powerful system in which to perform extensive genetic manipulations. Most cellular pathways are highly conserved from this simple eukaryote up to humans. Therefore, it was logical to develop yeast models of neurodegeneration in the hope to identify novel drug targets (Outeiro and Muchowski, 2004; Outeiro and Giorgini, 2006). In one such yeast model, expression of aSyn led inclusion formation and cytotoxicity. Importantly, cytotoxicity was developed by the simple duplication of the aSyn gene dosage, similarly to familial PD cases (Singleton et al. 2003; Outeiro and Lindquist, 2003). This yeast aSyn model was employed to screen for genetic suppressors or enhancers of toxicity (Willingham et al. 2003; Cooper et al. 2006). In a subsequent genetic screens heat shock proteins were identified as modulators of aSyn aggregation, and their roles in Lewy body formation were further validated in follow-up studies in mammalian cells (Outeiro et al. 2006; McLean et al. 2004; McLean et al. 2002).

These yeast models have also been used in high throughput compound screens as well. While yeast cells are known for poor compound uptake and for effective drug efflux systems that pump compounds out of the cell, mutations in the *ERG6* and *PDR1, 3,* and *5* genes, significantly improve the penetration and availability of small molecules. Compound screens have led to the successful identification of inhibitors of aSyn toxicity and aggregation, which might prove to have therapeutic application in the future (Griffioen et al. 2006).

### Mammalian cells

Phenotypic and functional PD assays were also developed using mammalian cells. The revolution in the field of RNA interferences greatly enhanced our ability to identify novel drug targets using screening platforms based on mammalian cells. For the transient gene knock-down in mammalian cells RNA duplexes (siRNA) or DNA, encoding RNA targeting duplexes, can be used. Viral-based delivery methods for the latter approach is broadly used, enabling a high percentage of infection of cells in culture (>90%), and hence to obtain uniform response (Berns et al. 2004; Raoul et al. 2006; Moffat et al. 2006). These methods can also be used to generate stable cell lines where the genes of interest are knocked down.

There is, however, a down-side to this potentially powerful approach. Cellular proteins are part of various complexes, involved in multiple cellular functions. Therefore, the downregulation of protein expression at the mRNA level will affect the activities of an indefinite number of protein complexes of which the individual target-protein is part of, with unpredictable consequences. Such putative pleiotropic effects on protein levels through single mRNA interference can lead to discrepancies between genetic and small molecule screening data.

High throughput platforms, based on phenotypic and functional readouts in mammalian cells, are widely used in drug screening of both synthetic compound and natural product libraries. A recent trend has been towards high content screening (HCS), when various features of cell morphology and functions are subjected to analysis. Such analysis potentially provides valuable evaluation for the effects of the drugs in the cell. However, due to extremely large number of data points generated in the HTS, the sizes of the screening libraries are quite limited at this point.

Nevertheless, advanced screening technologies provide opportunities for dissecting the cellular pathways involved in neurodegeneration and developing neuroprotective agents.

### Secondary and tertiary confirmatory assays

In the absence of a universal PD model that can recapitulate all features of pathology it appears difficult to properly evaluate the therapeutic potential of drug-candidates using a single model system. It is essential to validate and examine the properties of hits arising from primary screen in multiple secondary assays. Secondary assays are often low throughput and tedious, and are designed to test candidate molecules under physiological conditions. In PD, primary rat mesencephalic neuronal cultures, expressing virally delivered mutant (A53T) aSyn, are an example of a relevant secondary assay to validate hits modulating toxicity, aggregation and oligomerization of aSyn (Lo Bianco et al. 2002).

**Figure 2:**
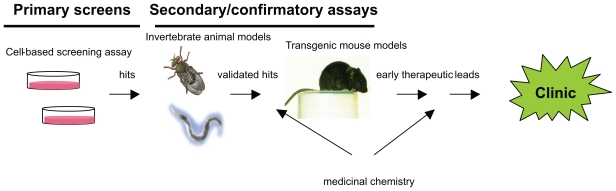
Drug discovery array describing the compound progression sequence from cell-based (black-box) assays through secondary, disease-specific, validation tests in other models organisms and, ultimately to the clinic.

Fruit fly and worm models of aSyn toxicity have also been developed over the years (Lo Bianco et al. 2002; Lakso et al. 2003; Auluck and Bonini, 2002; Feany, 2000). Despite apparent evolutionary differences between mammals and invertebrates, these animal models accurately recapitulated certain pathological features of neurodegeneration in PD. Therefore invertebrate animal models became an important intermediate step in the validation and assessment of therapeutic candidates, providing qualitative confirmation of agent efficacies for PD in the context of a whole animal (Fig. 2).

#### Rodent PD models

The ultimate test in the development of pre-clinical candidates is the demonstration of their efficacy in rodent disease models (Fig. 2). The development of accurate rodent PD models has faced several difficulties, due to inconsistencies in phenotypes and only partial recapitulation of disease features. Nonetheless, several PD models in mice and rats have been developed and used in the recent years for investigating the molecular pathogenesis involved in the disease (Masliah et al. 2000; Lo Bianco et al. 2002; St Martin et al. 2007).

Multiple transgenic mouse models of PD over-expressing wild-type and mutant forms of aSyn have been generated to help elucidate the role of the protein in the disease. Although none of the lines created display significant dopaminergic neuronal death in the substantia nigra, the models have specific attributes that recapitulate some features of synucleinopathies and are useful to study the potential pathogenic role of aSyn. In terms of widespread and robust aSyn aggregation, one of the best characterized lines is the line D aSyn over-expressers reported by Masliah et al. These mice develop an age-dependent aSyn aggregation within numerous neurons in the cortex and in the substantia nigra, as well as a detectable loss of striatal dopaminergic terminals.

To address the need for an animal model with specific nigrostriatal degeneration, researchers have employed gene therapy techniques. Viral vectors, including adeno-associated virus and lentivirus, have been successfully used to generate mouse, rat and nonhuman primate models with nigrostriatal degeneration by targeting aSyn expression to the substantia nigra. Along with recapitulating the cardinal pathological features of PD, these viral-based models provide a slow disease progression that more closely mimics human disease and allows for earlier points of characterization and/or intervention.

These rodent models provide novel opportunities to expedite drug development for therapeutic intervention in PD and other synucleinopathies.

## Conclusions

The primary pathological role proposed for aSyn oligomeric species in neurodegeneration is consistent with the growing number of experimental evidence. Although the molecular basis for oligomer neurotoxicity is still elusive, oligomeric species likely have a negative impact on the ubiquitin proteasome degradation machinery, and on mitochondrial function. These species also seem to trigger cellular defense pathways, such as the stress-response and caspase activation. The kinetics of oligomer formation is likely modulated by various cellular components, aging, and external stimuli, including oxidation. The identification of modifiers, capable of reducing oligomerization, is envisioned as a first priority for genetic screens. The oligomerization process can also be amenable to modulation by small molecules which are beginning to emerge in drug screens based on phenotypic and functional cell assays. Due to insufficient knowledge on the molecular targets modulating oligomerization, the evaluation of the therapeutic potential of the compounds identified in cell-based screens, may be tedious and not straightforward. A prudent experimental approach will be to assess their therapeutic values in genetic animal models. This concerted approach will ensure that the most promising leads can be moved forward towards human drug development irrespectively from their molecular mechanism of action.
